# Editorial: Update on glaucoma research: from basic science to clinical practice

**DOI:** 10.3389/fmed.2025.1572755

**Published:** 2025-02-27

**Authors:** Alessio Martucci, Maria Dolores Pinazo-Duran, Carlo Nucci

**Affiliations:** ^1^Ophthalmology Unit, Department of Experimental Medicine, University of Rome “Tor Vergata”, Rome, Italy; ^2^Ophthalmic Research Unit “Santiago Grisolia”, Foundation for the Promotion of Health and Biomedical Research of the Valencian Community, Valencia, Spain

**Keywords:** glaucoma, MIGS, tonometry, quality of life (QoL), microRNA (miRNA), acid uric levels, limbal stem cell deficiency (LSCD), blood pressure variability (BPV)

Glaucoma remains one of the leading causes of irreversible blindness worldwide. As an insidious disease often asymptomatic in its early stages, it poses unique challenges for timely diagnosis and treatment. This Research Topic sheds light on various aspects of glaucoma, ranging from factors leading to delayed diagnosis, innovations in intraocular pressure (IOP) measurement, the role of systemic health, neurodegenerative aspects and novel treatment approaches (1, 2).

In this context, a qualitative study by Liu et al. exploring factors associated with delayed ophthalmological consultation in primary glaucoma patients identified four major barriers: subtle onset of symptoms, insufficient patient awareness, perceived challenges in accessing care, and inadequate support systems. These findings underscore the urgent need for improved public health initiatives, patient education, and streamlined referral pathways to encourage early detection and prevent irreversible vision loss. Healthcare professionals should prioritize educating at-risk populations and integrating community-based screening programs to effectively address these barriers (Liu et al.).

Accurate IOP measurement is also critical to glaucoma management. In a comparative study between the Goldmann Applanation Tonometry and the SUOER SW-500 Rebound Tonometer, Chaung et al. revealed that the latter provides comparable readings within the normal IOP range but may underestimate elevated pressures. While rebound tonometry offers advantages such as portability and disposable probes to minimize infection risk, clinicians should remain cautious when relying solely on this method in patients with high IOP. Future research should explore the refinement of non-contact tonometry techniques to improve precision and reliability (Chaung et al.).

Novel biomarkers also offer a promising avenue for improving glaucoma diagnosis, prognosis, and treatment response assessment. By identifying molecular, genetic, and biochemical markers associated with glaucomatous neurodegeneration, clinicians may be able to detect the disease earlier, differentiate subtypes, and personalize treatment strategies. Biomarkers found in the aqueous humor, tear film, blood, and even imaging-based parameters could provide valuable insights into disease mechanisms and therapeutic targets (3).

In this context, given the neuroprotective role of uric acid (UA) in neurodegenerative diseases, its relationship with glaucoma has been the subject of investigation. A meta-analysis of multiple studies authored by Mohammadi et al. revealed that while glaucoma patients tend to have slightly higher serum UA levels than controls, the difference is not statistically significant. While these findings do not establish UA as a definitive biomarker for glaucoma, further longitudinal research is necessary to elucidate its potential role in the pathogenesis of the disease (Mohammadi et al.).

In a recent molecular research Yu et al. also identified apoptosis-related microRNAs (miRNAs) as potential biomarkers in glaucoma. Elevated levels of hsa-miR-193a-5p and hsa-miR-222-3p in aqueous humor and lens capsules suggest their involvement in glaucoma pathophysiology. The downregulation of PTEN, a key regulatory gene, further supports their role in retinal ganglion cell apoptosis. These findings pave the way for novel diagnostic and therapeutic strategies leveraging miRNA modulation (Yu et al.).

Evaluating local and systemic aspects is essential for comprehensive glaucoma management, as they may impact treatment response, disease progression, and overall patient outcomes. A multidisciplinary approach integrating ophthalmology, cardiology, neurology, and internal medicine may help optimize glaucoma care and preserve visual function.

As the incidence of glaucoma rises globally, patients require prolonged medical therapy and multiple surgeries, increasing the risk of systemic and local adverse events such as limbal stem cell deficiency (LSCD) risk.

In glaucoma, LSCD can be associated with multiple limbal surgeries, bullous keratopathy, mitomycin C, 5-fluorouracil, and preservatives in topical treatments. Using confocal microscopy and optical coherence tomography Nakakura et al. have demonstrated limbal epithelial thinning in glaucoma patients using topical medications.

Vascular dysregulation, systemic hypertension, hypotension, diabetes, obstructive sleep apnea, and neurodegenerative disorders have also all been implicated in the pathophysiology of glaucoma (4–6).

In this regard, a large population-based cohort study by Lee et al. examined the impact of visit-to-visit blood pressure variability (BPV) on the risk of open-angle glaucoma (OAG) in normotensive individuals. While BPV was not associated with an increased overall risk of OAG, younger individuals (< 60 years) with high systolic BPV had a significantly higher risk of developing the disease. These findings highlight the need for a more nuanced approach to monitoring cardiovascular and ocular health, particularly in younger patients (Lee et al.).

Furthermore, glaucoma not only affects visual function but also has a significant impact on the psychological wellbeing of patients. A study by Kopilaš and Kopilaš assessing quality of life (QOL) and psychological distress in glaucoma patients demonstrated strong correlations between disease progression, decreased visual acuity, and increased anxiety and depression.

Although observational studies have suggested a link between glaucoma and psychiatric conditions such as depression, insomnia, and schizophrenia, a Mendelian Randomization study by Zhang et al. and a study of anxiety in glaucoma by Lin et al. found no causal relationship between these conditions. These results indicate that psychiatric conditions in glaucoma patients are more likely to be due to modifiable factors rather than genetic predisposition.

The chronic nature of glaucoma, coupled with the gradual loss of vision, underscores the importance of holistic patient care. This highlights the need for targeted psychological support and intervention strategies rather than attributing mental health challenges to inherent disease risk. Mental health support should be integrated into routine glaucoma management to improve overall patient outcomes (Kopilaš and Kopilaš; Zhang et al.; Lin et al.).

Advancements in surgical techniques, such as Micro-invasive glaucoma surgery (MIGS), have improved the safety profile of glaucoma surgery, offering less invasive options with faster recovery times. Nevertheless, the selection of the appropriate surgical approach requires careful evaluation of patient-specific factors to optimize outcomes and minimize complications.

A recent retrospective study evaluated cases of ocular hypertension following EyeCee One preloaded intraocular lens (IOL) implantation. The findings indicated a significant increase in IOP in a subset of patients, emphasizing the importance of careful postoperative monitoring, especially in individuals with a history of glaucoma. González-Martín-Moro et al. suggest that clinicians should remain vigilant about potential IOP fluctuations and consider alternative IOL options where necessary.

MIGS has gained traction as a safer alternative to traditional surgery. A retrospective study by Hajduga-Szewczyk et al. evaluating iStent implantation in conjunction with cataract surgery demonstrated significant reductions in IOP and reliance on topical medications. However, patients with higher preoperative IOP showed limited benefit, suggesting that single iStent implantation may be insufficient in uncontrolled glaucoma cases. Further research to optimize MIGS techniques is warranted (Hajduga-Szewczyk et al.).

Recent advancements in glaucoma research highlight the multifaceted nature of the disease, spanning early detection, systemic influences, mental health considerations, and evolving treatment modalities. A concerted effort to improve patient education, refine diagnostic tools, integrate psychological support, and expand surgical options will be critical to mitigating the global burden of glaucoma. In the future, interdisciplinary collaboration among ophthalmologists, primary care physicians, mental health professionals, and researchers will be essential in developing a comprehensive, patient-centered approach to glaucoma care.
